# Gaining supervision skills in pre-registration nursing through peer teaching: An evaluative survey

**DOI:** 10.1016/j.heliyon.2022.e11398

**Published:** 2022-11-04

**Authors:** Karolina Martins, Amanda Wagg, Elsa Afonso

**Affiliations:** Anglia Ruskin University, United Kingdom

**Keywords:** Pre-registration nursing, Peer-led teaching, Student competencies, Student supervision

## Abstract

**Background:**

It is now a requirement that all qualified nurses act as practice supervisors and support student nurses' education in practice, hence preparing third-year students for this role is a priority. This study evaluates students' experiences of peer teaching in clinical skills setting from the perspective of these students taking up the supervisory role once they graduate.

**Method:**

An evaluative survey was utilised to explore and understand student nurse participation in peer teaching. Seventeen students took part in a questionnaire containing closed and open questions.

**Results:**

This research suggests that students who engaged in peer teaching gained confidence in their own skills, through the revision of their own skills and knowledge. It also triggered reflection upon continuous professional development and inspired students to consider a future career in teaching.

**Conclusion:**

Peer teaching provides an opportunity to reinforce the students' knowledge, clinical and communication skills. It helps prepare them for the role of practice supervisor upon qualifying by building confidence and enhancing their teaching skills.

## 1 Introduction

Peer teaching in pre-registration nursing education is a well-known concept already utilised in university skills laboratories (Stables, 2012), and clinical practice (Henderson et al., 2020). Topping (2005) defines peer learning as the transition of knowledge and skill through active support among learners of the same level. Similarly, near-peer teaching refers specifically to senior students teaching junior students from the same education program (McKenna and Williams, 2017). Research into peer teaching and near-peer teaching reports a range of positive outcomes for those students engaged in the process (Christiansen and Bell, 2010; George et al., 2020; Henderson et al., 2020; Loke and Chow, 2007; McKenna and French, 2011; Ramm et al., 2015), and there is an overall assumption that teaching a subject deepens the students' understanding for those who are in the teacher’s role (Biggs and Tang, 2011). Peer teaching, for example, in clinical skills, has been shown to increase self-efficacy (students gaining better understanding), decrease anxiety (being taught by peers) and contribute to cost-effectiveness, thus positively impacting a student’s learning (Brannagan et al., 2013).

During this near-peer teaching process, students also socially interact with each other and there is an opportunity for significant learning to occur. However, this is not conventionally recognised as knowledge acquisition within formal nursing education. This ‘hidden curriculum’, which could be described as the unintentional lessons learnt or the learning beyond the defined curriculum (McKenna and Williams, 2017), has been identified as playing an important role in student’s development of professional values and cultural competence as well as just skills acquisition (Paul et al., 2014). The concept of the hidden curriculum in nurse education is not new, yet near-peer teaching between senior and junior students is not well described (Irvine et al., 2018; McKenna and Williams, 2017).

The Nursing and Midwifery Council Standards for Student Supervision and Assessment (NMC, 2018a) state that every registered nurse in the United Kingdom will be responsible for student nurse learning in practice. This role will involve supervising and providing feedback to nursing students in clinical practice as soon as they become NMC registrants and start working. Providing experiences in which third-year nursing students can prepare for this role is therefore an important part of nurse training (McKenna and French, 2011; Ramm et al., 2015). For this reason, third year/final year nursing students were invited to support the clinical skills teaching of first-year nursing students in the university skills lab, and this evaluative project aimed to evaluate those experiences to identify any potential value/issues in this activity. This evaluative survey aims to better understand whether peer teaching, as part of the student nurse training, is of value to the students and to understand their perceptions on whether they feel it supports their future role.

## 2 Method

An evaluative survey using quantitative and qualitative data was utilised to explore the research. A questionnaire was chosen as a method of exploring the students' experiences, and best answer the research question. The questionnaire contained three parts: demographic/baseline data (e.g., age, gender, the programme of study, hours completed in teaching); five questions utilising a Likert scale (1–5); and five open questions that further explored the students' experiences, allowing participants the freedom to provide their responses (Table 1). To ensure the trustworthiness and rigour of the research tool, the questionnaire was piloted on two students prior, to ensure readability and student understanding.

**Table 1:** Survey items.

Part 1	Part 2	Part 3
Demographic questionnaire	Likert scale 1 = strongly disagree 2 = disagree 3 = neither 4 = agree 5 = strongly agree	Open questions
Gender: • Female • Male • Prefer not to answer/other Age: • 18–23 • 24–29 • 30–35 • 36–41 • 42–Over Programme of study: • Pre – registration Adult Nursing (BSc) • Pre – registration Adult Nursing (MSc) How many hours have you completed in teaching skills? • 3–9 • 10–16 • 17–22	1. I was fully prepared for the session. 2. I was clear of what was expected of me in the session. 3. I am now comfortable with supervising first year student nurses. 4. The first-year nursing students valued being taught by a third-year student nurse. 5. This innovative method of teaching should be compulsory for all third-year student nurses.	Why did you choose to participate in this teaching project? How did you prepare for the session/s? What have you learnt about yourself by participating in supervising first year student nurses? What have you enjoyed about supervising first-year student nurses? How, if any, has this experience of supervising prepared you for your future nursing career?

The invitation to participate was sent to all final year adult nursing students in one of the Higher Education Institutions in East of England (United Kingdom) but only thirty-three students took part in the study and volunteered to support a variety of clinical skills sessions for first-year nursing students. They all completed 3–22 h of near-peer teaching and were asked to either deliver part of the session or to supervise a small group of students completing a particular task within the session. This was agreed upon by the students before the session, based on their comfort level with the task at hand and all students were given any necessary resources a week in advance. All 33 students had an opportunity to practice the clinical skills prior to the teaching and had the mandatory lecture regarding the supervisor role in view of new NMC standards (NMC, 2018b).

The teaching project started in September 2018, ending in March 2020. Data was collated from June 2019 until August 2020 and of the 33 students taking part, 17 students returned the questionnaire, giving a response rate of 51.5 %. University ethical approval was obtained, and all responses were anonymously submitted either online or on paper.

The Statistical Package for the Social Sciences (SPSS 26) programme was used to analyse the quantitative data such as the demographic and Likert scale questions, using descriptive statistics. Open text questions (Table 1: column three) were thematically analysed using the Braun and Clarke method (2006). This is an inductive, iterative process of identifying patterns in the data. The primary researcher coded the qualitative data which was agreed by two other authors. This research set out to answer the following questions: Does peer teaching, as part of student nurse training, better prepare students for their inevitable role as a supervisor and if so, how?

## 3 Results

**Quantitative data:** The study included a total of 17 participants (3 males and 14 females), between 24 and 29 years old, and the majority were undertaking a BSc in adult nursing. 52.9% of the students had spent 17–22 h teaching peer skills, whilst 23.5% spent 3-9- or 10-16-hours teaching skills, as highlighted in Table 2. The majority spent greater than 10 h of peer teaching.

**Table 2:** Demographic characteristics of participants (N = 17).

Variable	N (%)
Study Sample	17 (100)
Gender	
Male	3 (17.6)
Female	14 (82.4)
Age group (years)	
18–23	4 (23.5)
24–29	5 (29.4)
30–35	1 (5.9)
36–41	4 (23.5)
42–48	3 (17.6)
Course	
Pre-registration Adult BSc	15 (88.2)
Pre-registration Adult MSc	2 (11.8)
Hours completed in teaching skills	
3–9	4 (23.5)
10–16	4 (23.5)
17–22	9 (52.9)

Students were asked to rate specific aspects of supervision and teaching preparation and expectations (Table 3). The majority agreed or strongly agreed that they felt sufficiently prepared for sessions and that the expectations were clear to them. When asked whether they were comfortable with teaching, the majority agreed or strongly agreed. However, 2 students responded that they were neither comfortable nor uncomfortable. To the question about whether first-year students valued being taught by third-year students, the majority replied that they either agreed or disagreed. Finally, when asked about whether teaching and supervision should be mandatory for third-year students, the results were more skewed. While half either agreed or strongly agreed, almost a quarter disagreed. One student strongly disagreed with all the questions. This was noted by researchers and may be attributed to non-conformity with the activity.

**Table 3:** Participants responses on Likert scale (N = 17).

Variable	Strongly disagree (n/%)	Disagree (n/%)	Neither agree nor disagree (n/%)	Agree (n/%)		Strongly agree (n/%)
I was fully prepared for the session	1 (5.9)	1 (5.9)	0	6 (35.3)		9 (52.9)
it was clear what was expected of me in the session	1 (5.9)	0	0	3 (17.6)		13 (76.5)
I am now comfortable with supervising first-year students	1 (5.9)	0	2 (11.8)	5 (29.4)		9 (52.9)
First-year students value being taught by third-year students	1 (5.9)	0	1 (5.9)	5 (29.4)		10 (58.8)
This innovative method of teaching should be compulsory for all third-year students	1 (5.9)	4 (23.5)	1 (5.9)	6 (35.3)		5 (29.4)

### 3.1 Qualitative data

Three themes emerged when analysing the open questions (Table 1). When asked to provide a narrative around their peer teaching the findings incorporated; their motivation to participate, the impact on their own learning, and the potential impact on their future careers. Overall students shared why they chose to participate and what it meant to them.

Theme one explored the motivation for participating in peer teaching. Amongst the motivations, an opportunity for revision was a key motivator. Students wanted to refresh their knowledge, work on their revision techniques, and keep up to date with any changes in clinical skills. They also expressed how the absence of peer teaching was a missed opportunity when they were first-year students themselves.

Theme two explored the impact of peer teaching on themselves, and confidence was a major sub-theme in this section. This peer teaching experience prompted self-reflection. They also alluded to the perception that by teaching others they were able to master their skills, and found it boosted their confidence: “now I’m able to teach and explain things, learnt how to engage with younger students; I knew more and have developed skills more than I had thought, feeling more confident”, and enjoyed knowing they had supported others: “opportunity to meet with junior; advise them, being able to answer questions about the course/my experience that a lecturer could not, the honest student experience, sharing my experience with them; seeing them learn, knowing that I have supported in that”. Another student noted that the peer teaching experience provided: “confidence to engage in teaching roles e.g., mentoring; I am more confident in teaching; I feel excited to work with students as a registered nurse; I will be confident in the new NMC model of assessing students on placement; a confidence to teach others and prepared me to teach junior/students when I qualify as nurse. I think this was very beneficial to my learning as a third-year student”. Students also wanted to have the opportunity to supervise others in the practice setting, stating: “It will better equip me for new NMC standards; gives me confidence to support student once qualified; give insight into what being a mentor might be like; gives confidence to teaching others and ability to share experience, knowledge and skills”.

The final theme that emerged was the perceived impact on their future nursing career. Students felt that it prompted continuous learning within the nursing profession, stating: “it prepared me to an endless learning process for the future; I’m more confident to engage in coaching but not without making sure first that my competencies are in place and my own practice is at a high standard”. Teaching project opened some possibilities to consider teaching in the professional role: “peer teaching offers career opportunities (teaching); allowed me to consider this for my future career; this will influence my future career”. One student stated: “I love to teach and feel passionate about this topic; enjoy teaching people, I will mention the participation in my job interview”.

## 4 Discussion

This evaluative survey set out to explore the experiences of student nurses undertaking peer teaching in a clinical skill setting. Findings suggest that peer teaching positively impacted the students, who found value in this activity in developing their own skills, experiences, and career pathway. Although there were egoistic motivations to better themselves, they also took value in helping others. This experience was, overall, seen to prepare them for their role as practice supervisor to new student nurses in the future.

Near-peer teaching for student nurses, in the practical skills' setting, is under-researched in the literature, but almost all current studies focus on the benefits (Dumas et al., 2015; George et al., 2020; Ramm et al., 2015; Zentz et al., 2014). Those exploring students' intentions of taking part in peer teaching have observed that knowledge consolidation, teaching preparation and the possibility of considering academia as a career opportunity were the main motivating factors (Irvine et al., 2019; Massy-Westropp et al., 2021) and something noted in this small research evaluation.

Participation in peer teaching has positively impacted the students themselves. Peer teaching enables them to expand their knowledge and skills, something also noted in the literature (Gregory et al., 2011). Gregory et al. (2011) recognised a significant increase in knowledge for peer teachers, compared to the students who only prepared for the sessions but did not participate in teaching. Our study suggests that peer teaching was seen as the opportunity to review skills and reflect on knowledge, and existing research had similar findings (Dumas et al., 2015; Goldsmith et al., 2006; Henderson et al., 2020; Stables, 2012). This also triggered reflection upon continuous professional development in the future, and deeper learning from reflection was especially seen in the literature (Loke and Chow, 2007; Ramm et al., 2015), as contributing to an increase in students' confidence (Christiansen and Bell, 2010; George et al., 2020; Loke and Chow, 2007; McKenna and French, 2011; Stables, 2012).

The peer teaching project was designed to support the students' transition into the supervisor role, a skill expected of them upon qualifying in line with the NMC future nurse standards (NMC, 2018b). It was expressed by the students in this study that such competency was developed, and they are ready to undertake the mentoring role in practice. This is not new and was also confirmed in other studies (Christiansen et al., 2011; Irvine et al., 2019; Ramm et al., 2015; Zentz et al., 2014). Overall, this study agrees with the literature in that, peer teaching allows students to consider the educator role in the future (Irvine et al., 2019; Stables, 2012) and therefore it influences their professional career.

McKenna and Williams (2017) have described the concept of the hidden curriculum in peer teaching, but further research is needed to explore the link between peer teaching and its social benefits, especially for peer teachers. This research goes part way in starting a further dialogue around this as students in their teaching role noticed that there is also learning happening through the socialisation processes. Such support and acting as role models can remarkably contribute to the development of professional values for junior students (Phillips, 2013). Discussion is warranted around whether such an intervention should be mandatory, and if all students would feel the same, as a small study this is uncertain at this time, and further larger studies are required to draw any generalisation. However, this study provides a starting block within the university to explore this further.

This teaching project was optional for students, so those that wanted to attend and participate did. Similarly, only a few studies had a formal teaching unit, mandatory to attend for the students. As the benefits are being seen across studies (Brannagan et al., 2013; Christiansen and Bell, 2010; Irvine et al., 2019; McKenna and French, 2011; McKenna and Williams, 2017), then discussion around embedding peer teaching into the curriculum is warranted. Furthermore, Roscoe and Chi (2007) have highlighted the need to support reflective knowledge-building in higher education students. Educators should be fostering activities that promote explaining and questioning rather than the simple transmission of knowledge (Roscoe and Chi, 2007).

This study was used to support the validation of the new pre-registration nursing curriculum programme, for 2020 in a UK-based University. It shaped the new programme, and it is now mandatory to attend the peer teaching sessions though it’s limited to the intended hours. This is structured into the classes in students' final module where they are prepared to teach and learn about the different teaching styles, the assessment process and giving feedback. They also must complete the University online Practice Supervisor course.

## 5 Conclusion

Benefits to peer-led skills teaching were suggested in this research, both actively and through the hidden curriculum suggested in the nursing literature. From the perspective of nursing students, peer teaching provides an opportunity to reinforce their knowledge, clinical and communication skills. It supports and prepares them for their role of practice supervisors upon registration, which is an NMC requirement. Other universities may benefit from introducing peer-led teaching into the curriculum to support this supervisory competency and skill.

## 6 Limitations

This is a small, single-setting study and this compromised the generalizability of findings. It could be interesting to perform similar research when peer teaching has become mandatory in nursing students' curriculum and there is formal preparation. Also, it would be beneficial to replicate the same study in clinical settings and compare findings. Future, wider research is needed to explore the phenomena of the hidden curriculum for peer teachers too.

This study had a low response rate (51.51%), which can also be viewed as a limitation. This is not unusual for the survey research method. Wang and Cheng (2020) noticed that using questionnaires to reach a large sample of the population of interest is relatively inexpensive but can result in low response rates. This low response can be due to a nonresponse bias, a systematic difference between responders (people who complete a survey) and non-responders (people who did not complete a survey), which is usually encountered in survey studies with mailed questionnaires (Wang and Cheng, 2020).

The response bias and social desirability could also influence the results and that those who chose to participate in the near-peer teaching may be more intrinsically motivated. Social desirability bias refers to the tendency to present oneself and one’s social context in a way that is perceived to be socially acceptable, but not wholly reflective of one’s reality (Bergen and Labonté, 2020). Many students have completed the questionnaire straight after the skills session finished which could affect rushing their answers. Therefore, their responses may have been not thoroughly considered. The same could happen with the questions, as some of them, focused only on the benefits of near-pear teaching and did not consider the negative side of this experience.

This research did not receive any specific grant from funding agencies in the public, commercial, or not-for-profit sectors.

## Declarations

### Author contribution statement

Karolina Martins: Conceived and designed the experiments; Performed the experiments; Analyzed and interpreted the data; Wrote the paper.

Amanda Wagg; Elsa Afonso: Analyzed and interpreted the data; Wrote the paper.

### Funding statement

This research did not receive any specific grant from funding agencies in the public, commercial, or not-for-profit sectors.

### Data availability statement

Data will be published and available at University ARRO system once the article is accepted.

### Declaration of interest’s statement

The authors declare no conflict of interest.

### Additional information

No additional information is available for this paper.

## Appendix A Supplementary data

Questionnaire Questionnaire
