# Supplementary material for: Gaining supervision skills in pre-registration nursing through peer teaching: An evaluative survey
Source: Heliyon. 2022 Nov 4;8(11):e11398. doi: 10.1016/j.heliyon.2022.e11398 (PMC9660580; doi:10.1016/j.heliyon.2022.e11398)
Supplement: Questionnaire [file mmc1.docx]

**Evaluating the participation of final year student nurses teaching clinical skills to first year student nurses.**

Main investigator: Karolina Filipiak, Clinical Skills Tutor (Adult Nursing), Anglia Ruskin University. Email: Karolina.Filipiak@anglia.ac.uk

Members of the research team: Dr Mary Edmonds, Senior Lecturer, Anglia Ruskin University.

Email: Mary.Edmonds@anglia.ac.uk

Dear Student,

You have been invited to participate in this research because you are a final year student who has taught clinical skills to first year nursing students. This research study intends to examine how and why nursing students benefit from teaching other students.

Before you make your decision, it is important for you to understand why the research is being done and what it will involve. Please take time to read the Participant Information Guidance which is attached with the questionnaire. Please check each of the statements below to confirm you are happy to participate. If you do, may I ask you to tick each of them and put a date. Consent will be implied by the return of this questionnaire.

1. I agree to take part in the above research. I have read the Participant Information Sheet (V2, 07/06/19) for the study. I understand what my role will be in this research, and all my questions have been answered to my satisfaction.

Date:

2. I understand that I am free to withdraw from the research without giving a reason, prior to completing a questionnaire and up to one week after the questionnaire has been sent to the main investigator, after which data cannot be withdrawn.

Date:

1. I am free to ask any questions at any time before and during the study.

Date:

1. I understand what will happen to the data collected from me for the research.

Date:

1. I have been provided with a copy of this form and the Participant Information Sheet.

Date:

1. I understand that quotes from me will be used in the dissemination of the research.

Date:

**Questionnaire**

**Part 1**

**Demographic questionnaire (please highlight/tick/cross the appropriate answer):**

Gender:

- Female
- Male
- Prefer not to answer

Age:

- 18–23
- 24–29
- 30–35
- 36–41
- 42–Over

Programme of study:

- Pre – registration Adult Nursing (BSc)
- Pre – registration Adult Nursing (MSc)

How many hours have you completed in teaching skills?

- 3-9
- 10 – 16
- 17 – 22

**Part 2**

**Each question will follow Likert scale: 1=strongly disagree 2=disagree 3=neither 4=agree 5=strongly agree**

**Please write the number which indicates your answer.**

**1. I was fully prepared for the session.**

1=strongly disagree 2=disagree 3=neither 4=agree 5=strongly agree

**2. I was clear of what was expected of me in the session.**

1=strongly disagree 2=disagree 3=neither 4=agree 5=strongly agree

**3. I am now comfortable with supervising first year student nurses.**

1=strongly disagree 2=disagree 3=neither 4=agree 5=strongly agree

**4. The first year nursing students valued being taught by a third year student nurse.**

1=strongly disagree 2=disagree 3=neither 4=agree 5=strongly agree

**5. This innovative method of teaching should be compulsory for all third year student nurses.**

1=strongly disagree 2=disagree 3=neither 4=agree 5=strongly agree

**Part 3**

**Open questions:**

**Why did you choose to participate in this teaching project?**

Provide comments:

**How did you prepare for the session/s?**

Provide comments:

**What have you learnt about yourself by participating in supervising first year student nurses?**

Provide comments:

**What have you enjoyed about supervising first year student nurses?**

Provide comments:

**How has this experience of supervising prepared you for your future nursing career?**

Provide comments:
